# Galectin-3 Overrides PTRF/Cavin-1 Reduction of PC3 Prostate Cancer Cell Migration

**DOI:** 10.1371/journal.pone.0126056

**Published:** 2015-05-05

**Authors:** Fanrui Meng, Bharat Joshi, Ivan Robert Nabi

**Affiliations:** Department of Cellular and Physiological Sciences, Life Sciences Institute, 2350 Health Sciences Mall, Vancouver, BC, V6T 1Z3, Canada; Institut Curie, FRANCE

## Abstract

Expression of Caveolin-1 (Cav1), a key component of cell surface caveolae, is elevated in prostate cancer (PCa) and associated with PCa metastasis and a poor prognosis for PCa patients. Polymerase I and Transcript Release Factor (PTRF)/cavin-1 is a cytoplasmic protein required for Cav1-dependent formation of caveolae. Expression of PTRF reduces the motility of PC3 cells, a metastatic prostate cancer cell line that endogenously expresses abundant Cav1 but no PTRF and no caveolae, suggesting a role for non-caveolar Cav1 domains, or Cav1 scaffolds, in PCa cell migration. Tyrosine phosphorylated Cav1 (pCav1) functions in concert with Galectin-3 (Gal3) and the galectin lattice to stabilize focal adhesion kinase (FAK) within focal adhesions (FAs) and promote cancer cell motility. However, whether PTRF regulation of Cav1 function in PCa cell migration is related to Gal3 expression and functionality has yet to be determined. Here we show that PTRF expression in PC3 cells reduces FAK stabilization in focal adhesions and reduces cell motility without affecting pCav1 levels. Exogenous Gal3 stabilized FAK in focal adhesions of PTRF-expressing cells and restored cell motility of PTRF-expressing PC3 cells to levels of PC3 cells in a dose-dependent manner, with an optimal concentration of 2 µg/ml. Exogenous Gal3 stabilized FAK in focal adhesions of Gal3 knockdown PC3 cells but not in Cav1 knockdown PC3 cells. Cav1 knockdown also prevented Gal3 rescue of FA-associated FAK stabilization in PTRF-expressing PC3 cells. Our data support a role for PTRF/cavin-1, through caveolae formation, as an attenuator of the non-caveolar functionality of Cav1 in Gal3-Cav1 signalling and regulation of focal adhesion dynamics and cancer cell migration.

## Introduction

Caveolin-1 (Cav1), a member of the caveolin protein family, is a key component of caveolae, the flask-shaped invaginations on the cell surface involved in many cellular processes such as vesicular transport, intracellular signaling and mechanical transduction [1]. Cav1 is involved in regulation of lipid rafts and of multiple cancer-associated processes including cell death and survival, cell migration and invasion, and tumor growth and metastasis [1–4]. Cav1 expression is elevated in metastatic prostate cancer (PCa) cells and Cav1 has been evaluated as a prognostic marker of aggressive PCa [5–7]. Cav1 has also been found associated with PCa metastasis in mouse and human PCa cell lines [5, 8]. PC3 is a metastatic prostate cancer cell line that expresses abundant levels of tyrosine phosphorylated Cav1 (pCav1) but lacks cell surface caveolae due to the absence of polymerase 1 and transcript release factor (PTRF)/cavin-1, required together with Cav1 for caveolae formation [7, 9–11]. Overexpression of PTRF in PC3 cells decreases cell motility via reduced matrix metalloprotease 9 (MMP9) production [12]. Further studies have shown that PTRF/cavin-1 expression alters the PC3 cell secretome by affecting cholesterol dynamics and the actin cytoskeleton, and attenuates promotion of PCa progression by non-caveolar Cav1 microdomain [13, 14].

Cell migration, a critical element of metastatic disease, is a dynamic and multistep process regulated through spatiotemporal feedback between actomyosin contraction, actin polymerization, and continuous disassembly and formation of adhesions [15–17]. Focal adhesions (FAs) are macromolecular assemblies that link the extracellular matrix and the cytoskeleton and transmit mechanical force and regulatory signals [18, 19]. Focal adhesion kinase (FAK) is the major kinase involved in FA signaling and regulates focal adhesion dynamics through its kinase domain (FRNK) and tyrosine 397 (Y397) autophosphorylation. Reduced FAK Y397 phosphorylation is associated with increased FAK exchange between FAs and cytosol, slower FA disassembly and reduced cell migration [20, 21]. pCav1 increases membrane lipid order in FAs and promotes FAK stabilization within FAs in multiple cancer cell lines including the PC3 PCa cell line, a cell line that expresses no endogenous PTRF and thus no caveolae, suggestive of a role for non-caveolar Cav1 scaffolds [4, 11, 22]. Cav1 is a major substrate of Src kinase and is phosphorylated on tyrosine 14 (Y14) [23–26]. In human breast, colon and prostate cancer cell lines, Src-dependent phosphorylation of Cav1 promotes FAK stabilization in focal adhesions, focal adhesion turnover, RhoA signaling and cell migration and invasion [11]. Galectin-3 (Gal3), a galactose-specific lectin family that preferentially binds cell surface GlcNAc-transferase V (Mgat5)-modified N-glycans, stimulates FAK and PI3K activation, enhances integrin activation and recruitment to fibrillar adhesions, and increases F-actin turnover [27–30]. We have shown that pCav1 and Gal3 act together to stabilize FAK within FAs and promote FA dynamics and cell migration and that coordinate expression of Cav1 and Gal3 distinguish differentiated thyroid cancer from benign [4, 31].

However, it has yet to be determined whether and how expression of PTRF and, thereby, caveolae, impacts the concerted Cav1-Gal3 regulation of FA dynamics and cell migration. We therefore used PC3 PCa cells that lack PTRF and caveolae but express Cav1 and pCav1, to specifically determine the role of PTRF in Cav1-Gal3 regulation of FA dynamics and cancer cell migration.

## Results

### Expression of PTRF/Cavin-1 decreases PC3 cell migration and disrupts FAK stabilization in focal adhesions

**Fig 1:**
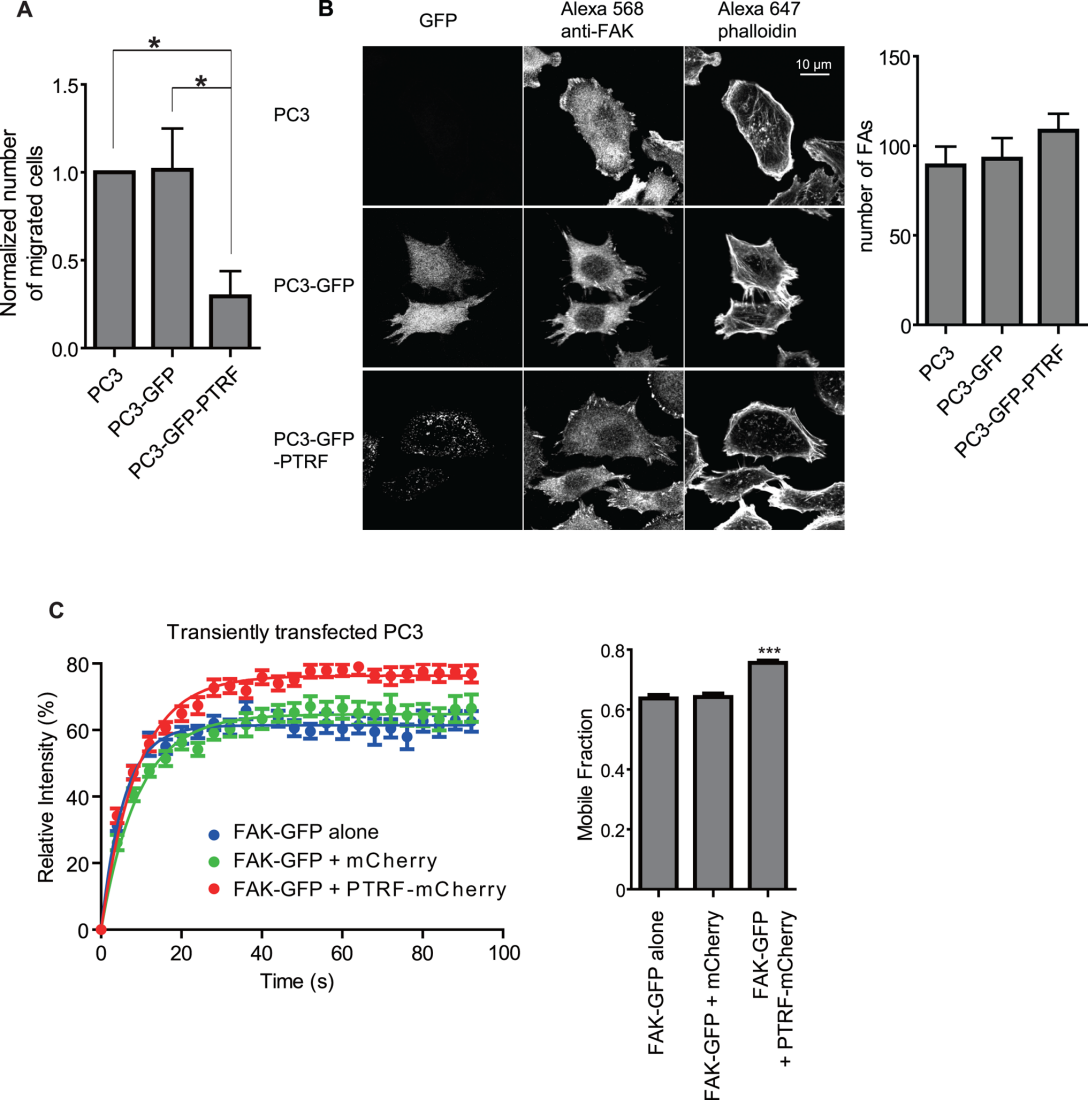
Expression of PTRF in PC3 cells reduces cell motility and FAK stabilization in FAs. (A) Transwell migration assay shows that PC3-GFP-PTRF cells migrate slower than wild-type PC3 and PC3-GFP cells. (B) Representative confocal images of PC3, PC3-GFP and PC3-GFP-PTRF cells and quantification of FAs per cell in each of the cell lines show that the number of FAs per cell is not significantly affected by PTRF expression in the cell. (C) Fluorescence Recovery After Photobleaching (FRAP) assay shows that FAK-GFP intensity recovery level is increased with PTRF-mCherry co-transfection compared with FAK-GFP alone or FAK-GFP and mCherry co-transfection. The FAK-GFP intensity recovery curve graph of one representative experiment and a bar graph of the FAK-GFP mobile fraction (calculated based on the fluorescence recovery plateau) summarized from all experiments are shown. (n≥3; ***: p<0.001; **: p<0.01; *: p<0.05.)

PC3 prostate cancer cells stably expressing PTRF-GFP (PC3-GFP-PTRF) show decreased cell motility compared with PC3 cells stable expressing GFP (PC3-GFP) or non-transfected PC3 cells (Fig 1A), as previously reported [12]. The reduced migration of PC3-PTRF-GFP cells was not associated with an altered number of focal adhesions labeled for FAK (Fig 1B). To determine FAK stability in FAs, PC3 cells were transfected with FAK-GFP alone, FAK-GFP plus mCherry or FAK-GFP plus PTRF-mCherry and subjected to fluorescence recovery after photobleaching (FRAP) assay. As shown in Fig 1C, the expression of PTRF-mCherry increased the mobile fraction of FAK-GFP in FAs relative to PC3 cells transfected with FAK-GFP alone or FAK-GFP with mCherry. An increased mobile fraction, increased fluorescence recovery of FAK-GFP relative to the pre-bleach fluorescence intensity, reflects higher exchange of FAK-GFP between the focal adhesion and cytosol hence less stabilization of FAK-GFP within FA. PTRF expression therefore diminished FAK stabilization in FAs, indicative of increased exchange with cytoplasmic FAK and reduced FA disassembly [20, 21].

### PTRF expression does not affect PCAV1 levels but additional GAL3 treatment restrores FAK stabilization in the FA and cell motility of PTRF-expressing PC3 cells

**Fig 2:**
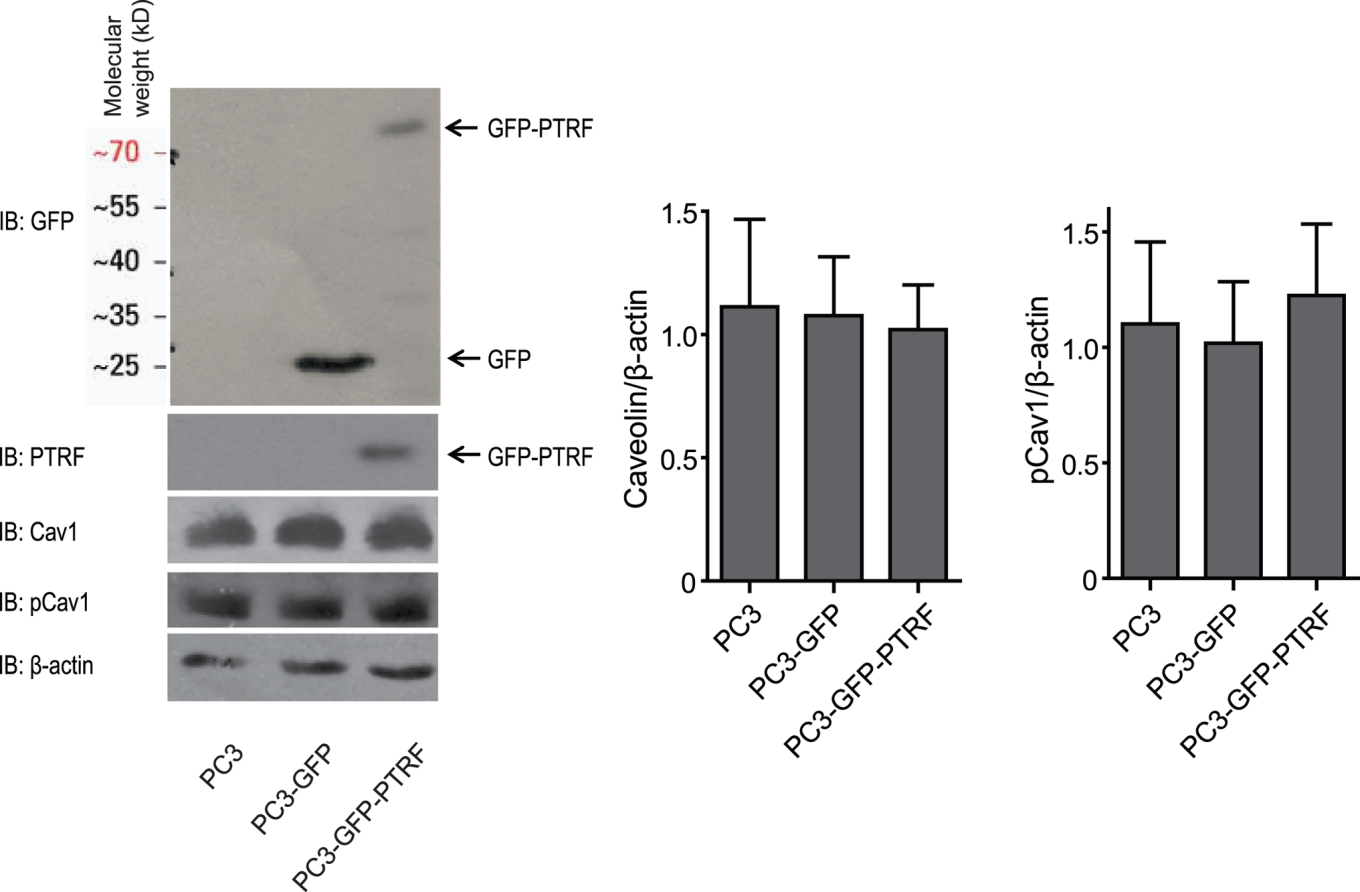
Expression of PTRF does not affect pCav1 in PC3 cells. Western blot shows expression levels of GFP, GFP-PTRF, Cav1 and pCav1 in PC3 wild-type cells (PC3), PC3 cells stably transfected with GFP (PC3-GFP) and PC3 cells stably transfected with GFP-PTRF (PC3-GFP-PTRF). Western blot band intensity of Cav1 and pCav1 is quantified and normalized to that of β-actin and shows no significant difference of Cav1 expression or phosphorylation between PC3, PC3-GFP and PC3-GFP-PTRF cells. (n≥3; ***: p<0.001.)

**Fig 3:**
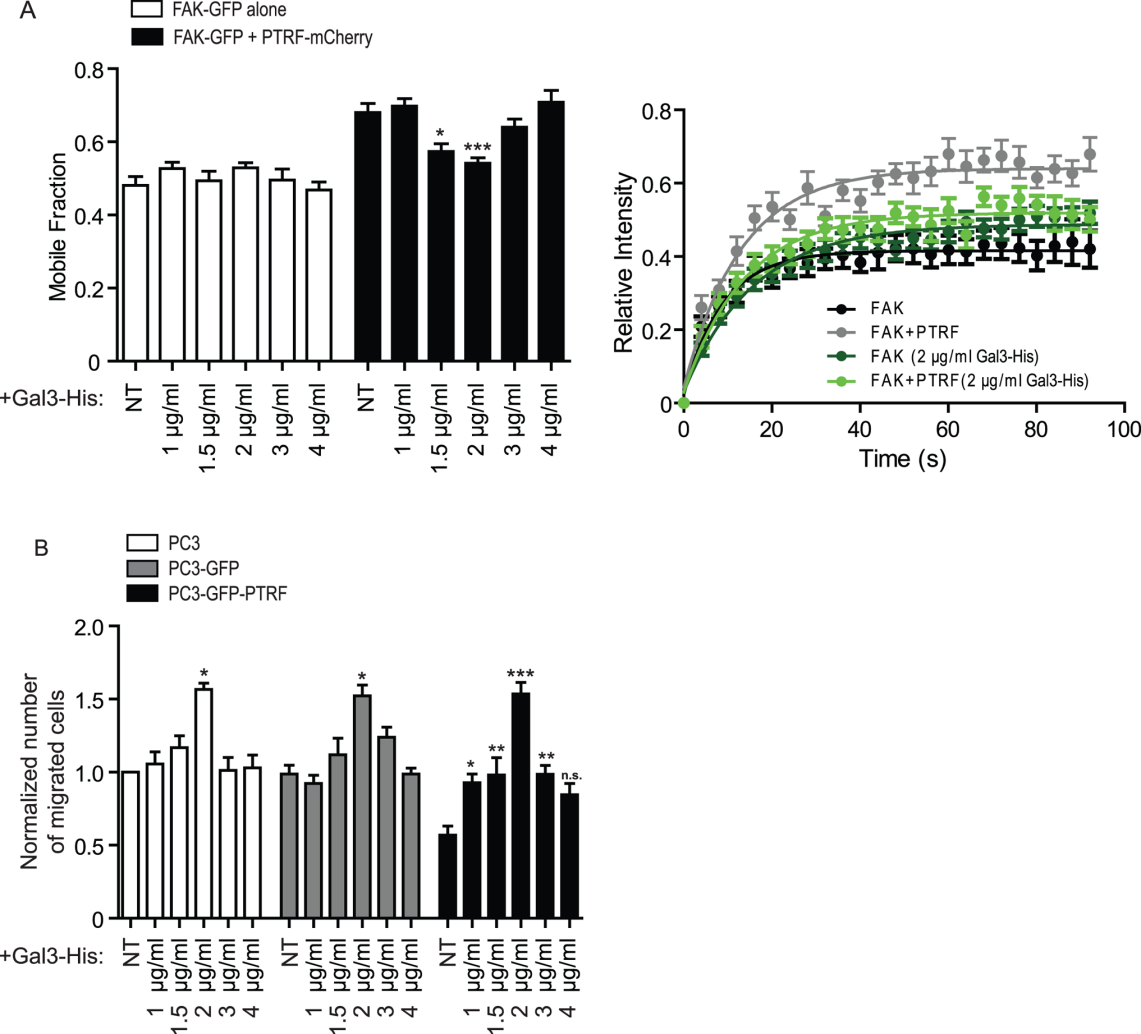
Exogenous Gal3 restores FAK stabilization in FAs and cell migration of PTRF-expressing PC3 cells in a dose-dependent manner. (A) FRAP assay of FAK-GFP shows the FA-associated FAK stability in PC3 cells transfected with FAK-GFP alone or FAK-GFP plus PTRF-mCherry and subjected to Gal3-His treatment at the indicated concentrations (1, 1.5, 2, 3, or 4 μg/ml). Reduced FAK stabilization in FAs of PTRF-expressing PC3 cells is restored by Gal3-His in a dose-dependent manner. A bar graph of the FAK-GFP mobile fraction summarized from all experiments and a representative FAK-GFP recovery curve graph from one experiment (showing NT and 2 μg/ml Gal3-His treatment only) are shown. (B) Quantification of PC3, PC3-GFP and PC3-GFP-PTRF cell migration using the transwell assay with no treatment or treated with Gal3-His (1, 1.5, 2, 3 or 4 μg/ml) shows that 2 μg/ml Gal3-His treatment increases the cell migration of all cell lines to a similar extent. Other concentrations of Gal3 increase migration of PC3-GFP-PTRF cells to a lesser extent, but do not affect migration of PC3 or PC3-GFP cells. (NT: non-treated; n≥3; *: p<0.05; **: p<0.01; ***: p<0.001; n.s.: not significant.)

Tyrosine-phosphorylated Cav1 (pCav1) regulates the migration of multiple cancer cell lines including PC3 [4, 11]. Stable PTRF expression in PC3 cells, however, did not alter Cav1 expression levels or phosphorylation (Fig 2). As Gal3 has been shown to function together with pCav1 to regulate FAK stability in FAs [4, 31], we tested whether exogenous Gal3 could restore FAK stabilization in FAs in PC3-PTRF-GFP cells. Addition of His-tagged Gal3 (Gal3-His) at concentrations from 1–4 μg/ml did not affect FAK stabilization within FAs in PC3 cells. However, in PTRF-expressing PC3 cells, addition of 1.5 or 2 μg/mlGal3-His significantly restored the stabilization of FAK in FAs, with 2 μg/ml of Gal3-His stabilizing FAK in FAs most significantly (p<0.001); addition of higher (3 or 4 μg/ml) or lower (1 μg/ml) concentrations of Gal3-His failed to restore the FA associated FAK stabilization (Fig 3A). The dose-dependence of the Gal-His stabilization of FAK in FAs is consistent with the concept of the galectin lattice, in which a critical ratio of Gal3 to glycan substrates enables optimal glycoprotein crosslinking that leads to receptor dynamics and signaling [32, 33]. A Transwell cell migration assay showed that the optimal 2 μg/ml Gal3-His enhanced the migration of all three cell lines (PC3, PC3-GFP and PC3-GFP-PTRF) to the largest extent and to the same level (Fig 3B). 1, 1.5 and 3 μg/ml of Gal3-His had no effect on the cell migration of control PC3 and PC3-GFP cells, but elevated PC3-GFP-PTRF cell migration to the level of untreated PC3 and PC3-GFP cells (Fig 3B). 4 μg/ml Gal3 did not significantly impact cell migration of any of the cell lines (Fig 3B). Addition of exogenous Gal3 protein therefore restores the deficient FAK stabilization in FAs and the reduced migration of PTRF-expressing PC3 cells in a dose-dependent manner.

### GAL3 rescue of FA-associated FAK stabilization is CAV1-dependent

**Fig 4:**
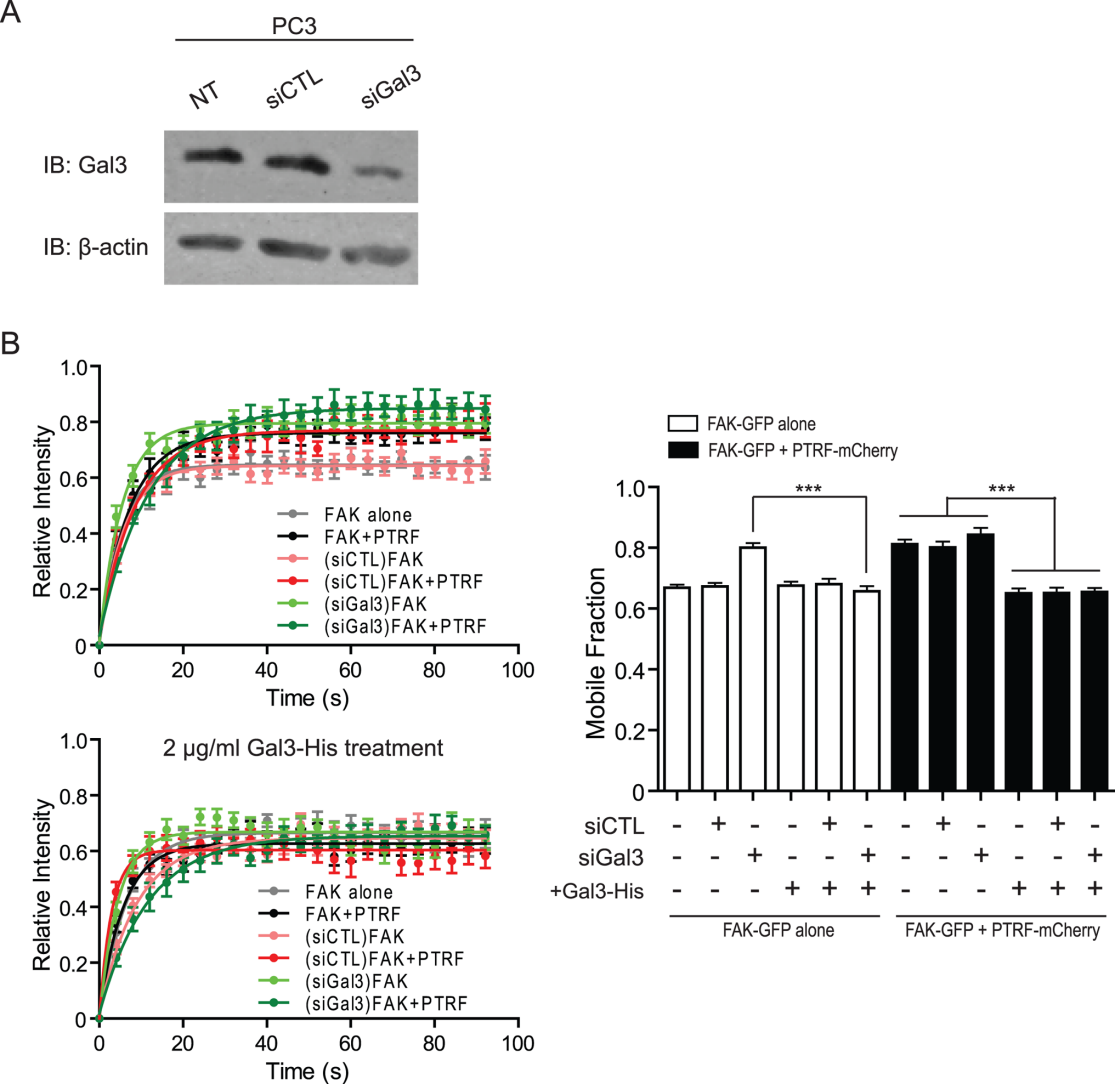
Exogenous Gal3 restores FAK stabilization in FAs of Gal3 knockdown cells. (A) Western blot shows the efficiency of Gal3 siRNA knockdown. (B) FRAP assay shows FA-associated FAK-GFP stability in PC3 cells transfected with no siRNA, scramble control siRNA (siCTL) or the siRNA against human Gal3 (siGal3), and subjected to 2 μg/ml Gal3-His treatment. The FAK-GFP intensity recovery curve graphs of one representative experiment and a bar graph of the FAK-GFP mobile fraction summarized from all experiments are shown. (n = 3; ***: p<0.001.)

To determine whether endogenous Gal3 stabilizes FAK in FAs of PC3 cells, we depleted Gal3 with siRNA obtaining a consistent 90% reduction in Gal3 levels after 48 hours (Fig 4A). After 24 hours we transfected the cells with FAK-GFP alone or FAK-GFP together with PTRF-mCherry, and treated them or not with 2 μg/ml Gal3-His prior to FRAP analysis of FAK-GFP stability in FAs. As shown in Fig 4B, knockdown of Gal3 (Gal3 KD) reduced FAK stabilization in FAs, to a similar extent as observed for PTRF-expressing PC3 cells, while siCTL had no effect on FAK stabilization compared to untransfected cells. Importantly, treatment with Gal3-His rescued FAK stabilization in FAs in both PTRF-expressing and Gal3 knockdown cells (Fig 4B).

**Fig 5:**
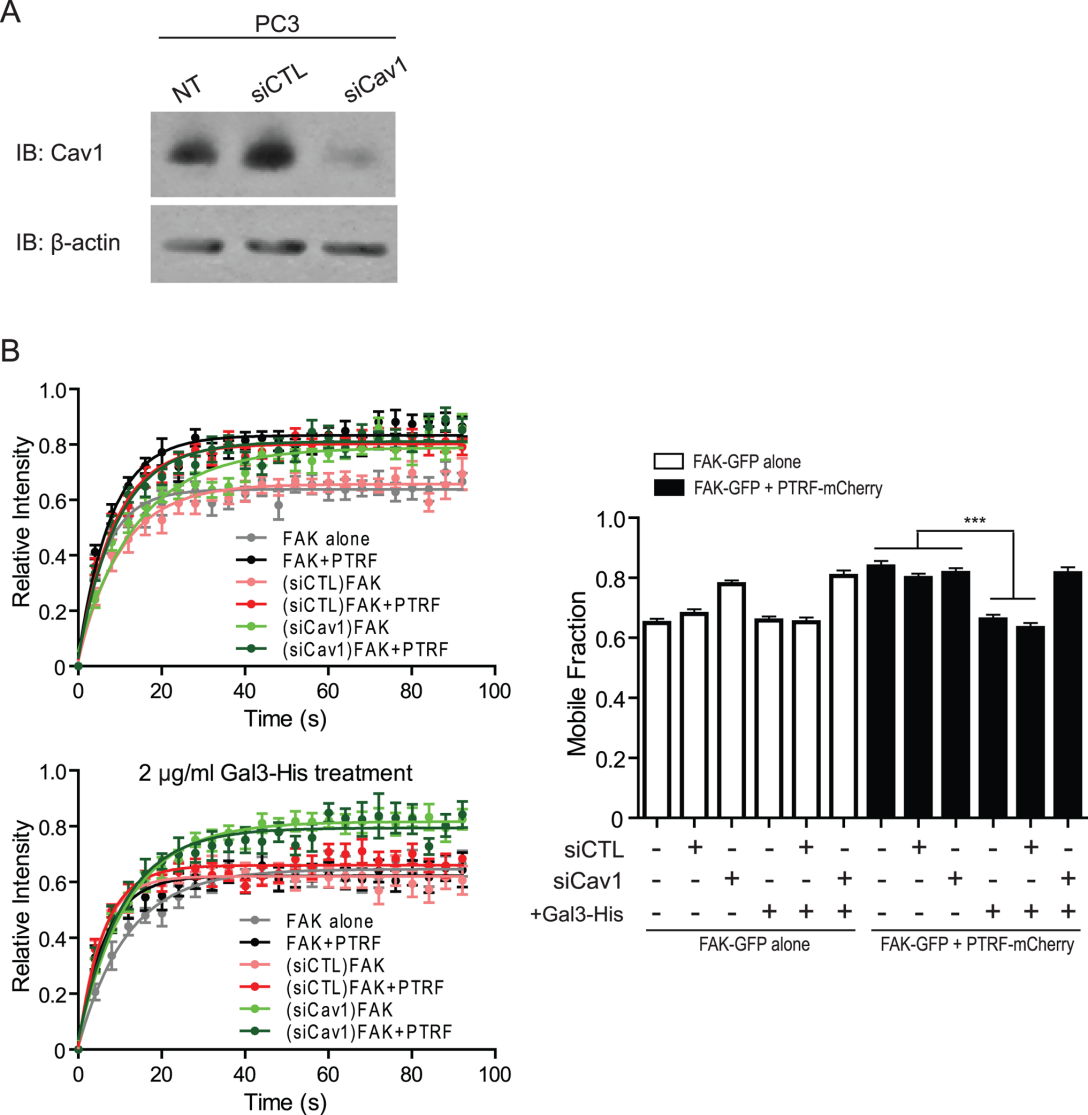
Exogenous Gal3 does not restore FAK stabilization in FAs of Cav1 knockdown cells. (A) Western blot shows the efficiency of Cav1 siRNA knockdown. (B) FRAP assay of FAK-GFP shows FA-associated FAK stability of PC3 cells transfected with no siRNA, scramble control siRNA (siCTL) or the siRNA against human Cav1 (siCav1), and subjected to 2 μg/ml Gal3-His treatment. The FAK-GFP intensity recovery curve graphs of one representative experiment and a bar graph of the FAK-GFP mobile fraction summarized from all experiments are shown. (n = 3; ***: p<0.001).

We then knocked down Cav1 in PC3 cells by siRNA (siCav1) (Fig 5A) before transfection with either FAK-GFP alone or FAK-GFP plus PTRF-mCherry and treatment with exogenous Gal3-His (2 μg/ml). siCav1 increased the mobile fraction of FAK-GFP in FAs in PC3 but not in PTRF-expressing PC3 cells, demonstrating that Cav1 selectively regulates FA dynamics in PC3 cells lacking PTRF (Fig 5B). Gal3-His was unable to promote FAK stabilization in FAs of PC3 or PC3-PTRF cells in which Cav1 was knocked down (Fig 5B). Cav1 is therefore necessary for Gal3-dependent FAK stabilization in FAs of PC3 cells. Concerted regulation of FA dynamics by Cav1 and Gal3 is therefore impacted by expression of PTRF and Cav1 recruitment to caveolae. This suggests that recruitment of Cav1 to caveolae may alter the relationship between non-caveolar Cav1 and Gal3 that is critical to their coordinated regulation of FA dynamics and cancer cell migration.

## Discussion

The cavin family includes PTRF/cavin-1, SDPR/cavin-2, PRKCDBP/cavin-3 and MURC/cavin-4, which share a conserved N-terminal domain comprised of heptad repeats of hydrophobic amino acids, possibly forming coiled coils, and a following region of several basic amino acids [34]. Expression of the cavins is associated with the expression and stabilization of Cav1 and the formation and function of caveolae [34]. Of the cavins, PTRF/cavin-1 is essential for caveolae formation and loss of PTRF results in loss of caveolae and downregulation of Cav1 protein levels [9, 35]. Cav1 has been ascribed non-caveolar functions [36–38] and stoichiometry between caveolins and cavins must necessarily influence the expression of not only caveolae but also non-caveolar Cav1 domains or Cav1 scaffolds. PC3 cells are an excellent cellular model to study non-caveolar functions of Cav1 as they lack PTRF and caveolae but show elevated levels of Cav1 [7]. Indeed, expression of PTRF in PC3 cells neutralizes the non-caveolar Cav1 microdomains and alters the cell motility, secretion pathways and angiogenesis and lymphangiogenesis abilities in PC3 cells [12–14, 39, 40]. Consistently, we show here that PTRF expression in PC3 cells limits Cav1-Gal3 regulation of FAK stabilization in FAs and cell motility.

Gal3 and pCav1 function in concert to promote FA dynamics and cell motility. pCav1 promotes cell polarization and migration and regulates membrane lipid order at FAs [4, 22, 26]. Gal3 binding to cell surface Mgat5-modified N-glycans forms a galectin lattice that stimulates FAK and PI3K activation, and promotes integrin activation and F-actin turnover [27–30]. Gal3 induces the integrin α5β1 activation and FAK (p397FAK) phosphorylation, associated with FAK stabilization in FAs, increased FA signaling and disassembly, and hence cell migration [20, 21, 28]. Furthermore, in cells lacking the galectin lattice due to absence of Mgat5, pCav1 is not sufficient to promote FA-associated FAK stabilization; expression of Mgat5 and the galectin lattice increases cell spreading and induces FAs but requires pCav1 to stabilize FAK within FAs [4]. Consistently, coordinated expression of Cav1 and Gal3 is observed in differentiated thyroid cancer-derived cell lines and siRNA knockdown assays in the same cell lines show that both Cav1 and Gal3 are required to promote FAK stabilization in FAs and cell migration compared to benign thyroid lesion-derived cells [31]. More recent data showing that Gal3 is required for epidermal growth factor (EGF) signaling that induces Cav1 phosphorylation and promotes circular dorsal ruffle (CDR) formation, cell migration and fibronectin fibrillogenesis; these studies led to the suggestion of a Gal3-integrin-pCav1 signalling module that mediates EGF signaling to Rho A [41]. This suggests that outside-in integrin signaling mediates concerted Gal3-pCav1 regulation of FA dynamics, where extracellular Gal3 interacts with and activates integrins which then recruits pCav1 leading to downstream RhoA signaling and cell migration.

**Fig 6:**
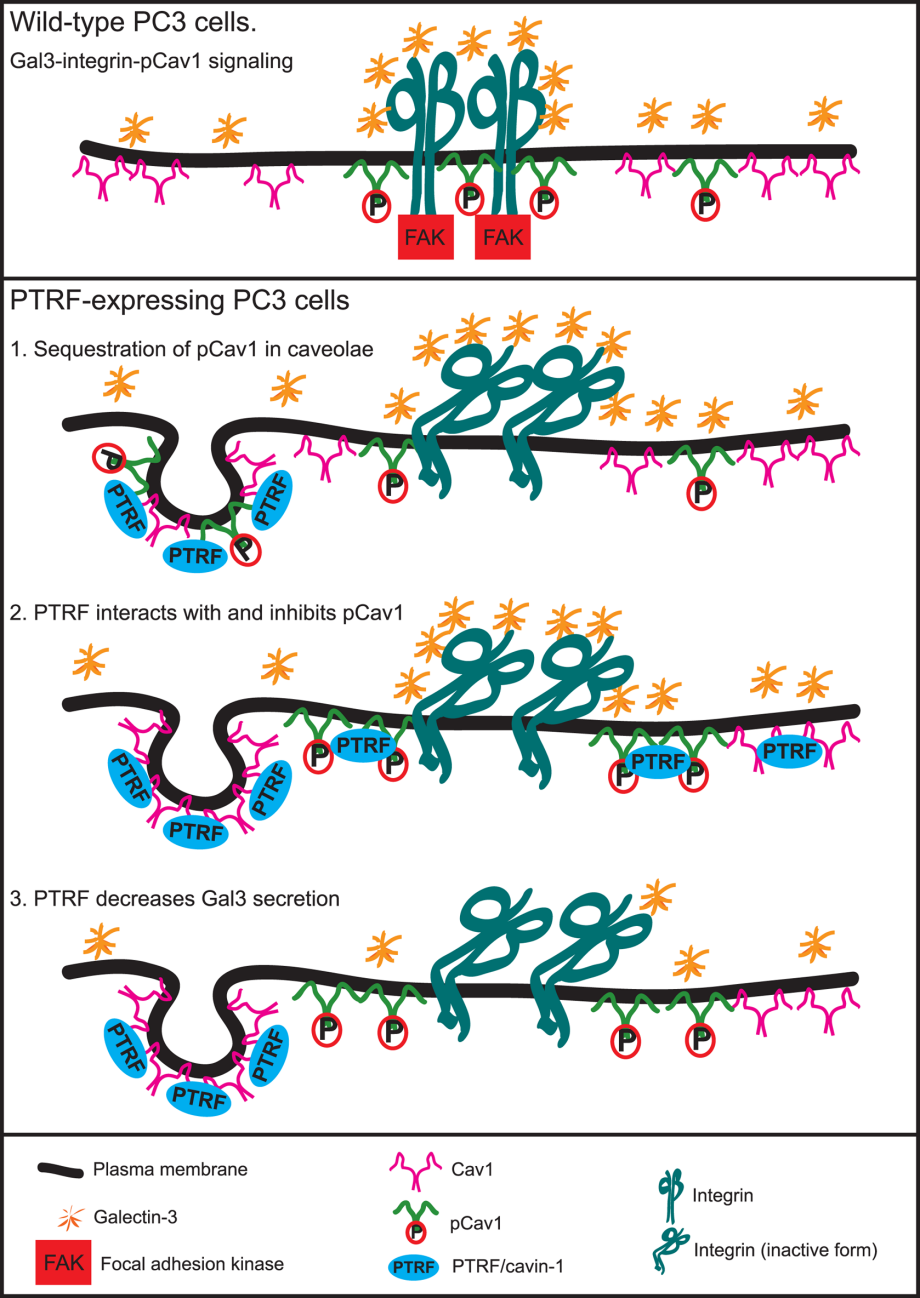
Summarized working models of affecting Gal3-pCav1 function. Extracellular Gal3 and non-caveolar pCav1 each stabilizes FAK within FAs dependent on each other. Expression of PTRF disrupts this function through three possible ways: 1) by recruiting pCav1 away from non-caveolar Cav1 scaffolds and into caveolae; 2) by direct interaction with non-caveolar pCav1; 3) by affecting Gal3 secretion and thus extracellular Gal3 concentration. Through which pathway PTRF affects Gal3-pCav1 function on FA dynamics remains to be studied.

Our data shows that PTRF expression in PC3 cells does not alter Cav1 or pCav1 levels, which suggests that PTRF regulates pCav1 function in FA dynamics through Cav1 recruitment to caveolae, reducing availability of functional pCav1 in non-caveolar scaffold domains. Formation of caveolae may regulate the expression and function of non-caveolar Cav1 scaffolds by recruiting pCav1 to caveolae. Cav1 Y14 phosphorylation is dispensable for caveolae formation but pCav1 has been localized to caveolae [42, 43]. Indeed, it has been shown that preferential expression of Cav1 in non-caveolar Cav1 domains due to an absence of PTRF is associated with advanced PCa and that expression of PTRF/caveolae neutralizes the non-caveolar Cav1 domains to slow down PCa progression [14]. The fact that excess Gal3 can restore pCav1-dependent FA signalling indicates that concerted interaction between Gal3 and pCav1 is critical to their ability to regulate FA signaling and dynamics. Of a range of concentrations from 1–4 μg/ml, 2 μg/ml was optimal to rescue PTRF-expressing cells, highlighting the concentration dependence of Gal3 lattice function. Similarly, Gal3 stimulation of integrin-dependent fibronectin fibrillogenesis is concentration dependent [28]. Integrins are modified by Mgat5 and Gal3 activates α5β1 integrin and recruits it to fibrillar adhesions [28, 44]. pCav1 has been shown to interact with integrins and modulate lipid order in FAs [22, 45–47]. Our data suggests that the relative concentration of Gal3 to non-caveolar pCav1 is a critical regulator of Gal3-pCav1 signaling and that PTRF is a novel regulator of this signaling module. PTRF has been shown to alter exosomal secretion of PC3 cells [13] and whether PTRF disrupts the Gal3-pCav1 module by limiting Gal3 secretion, or by sequestering pCav1 into caveolae and away from non-caveolar Cav1 scaffolds, or even by directly interacting with non-caveolar Cav1 scaffolds, remains to be determined (Fig 6).

Both Cav1 and Gal3 play important roles in PCa progression. Gal3 has been recognized as an important regulator of PCa metastasis [48–50] and several attempts have been performed to target Gal3 in PCa, using either the Gal3-binding cancer-associated Thomsen-Friedenreich glycoantigen (TF-Ag)-mimic lactose-L-leucine or a high-affinity Gal3-bingding glycopeptide purified from cod [51, 52]. Cav1 has been evaluated as a prognostic marker of aggressive PCa since the 1990s [5, 6].

More recent data suggests that PTRF neutralizes Cav1 action implicating a critical role for non-caveolar domains in PCa [14]. Our demonstration that Gal3 can reverse the reduced FAK stabilization in FAs and cell motility induced by PTRF identifies Gal3 and pCav1 as critical regulators of the function of non-caveolar domains in PCa cell migration. Coordinate activity of Cav1 and Gal3 may therefore play an important role in PCa metastasis and represent a potential therapeutic target for PCa.

## Materials and Methods

### Antibodies, plasmids, siRNA an recombinant human Gal3-His

Bovine serum albumin (BSA), and mouse anti-β-actin antibodies were purchased from Sigma-Aldrich. Rabbit anti-FAK was purchased from Santa Cruz Biotechnology, Inc., rabbit anti-pY14Cav1 from Cell Signaling, Inc., and mouse anti-PTRF and rabbit anti-Cav antibodies from BD Transduction Laboratories. HRP-conjugated mouse and rabbit secondary antibodies were purchased from Jackson ImmunoResearch Laboratories. Phalloidin and secondary antibodies conjugated to Alexa 488, 568, or 647 were purchased from Life Technologies, Thermo Fisher Scientific. PTRF-mCherry and mCherry plasmids were generous gifts from Dr. Michelle Hill (The University of Queensland Diamantina Institute, The University of Queensland, Translational Research Institute, Brisbane, QLD, Australia). FAK—GFP plasmid was as described previously [11]. Validated ON-TARGET plus SMARTpool small interfering RNAs (siRNA) for human Cav1, control siRNA (siCONTROL non-targeting siRNA no. 1) and custom siRNA duplexes for Gal3 [53] were purchased from Dharmacon.

Recombinant human Gal3 tagged with 6XHis at C-terminal was generated using the plasmid, pHIS-Parallel2, described previously [54]. This plasmid and protocols were a kind gift from Ludger Johannes (Institut Curie, Paris, France). In order to generate Gal3-His, we used BL21-DE3 bacteria (New England Biolabs). Briefly, overnight culture was re-inoculated (1:5) in fresh LB supplemented with ampicillin and cultured at 37 degree centigrade on 225 rpm shaker till optical density at 600 reached 1 and then induced using 0.4 mM IPTG for 3 h at 30 degree centigrade on a 225 rpm shaker. Recombinant Gal3-His was purified on Talon affinity matrix (Clontech) using the supplied protocol, transferred to 1xPBS prior to quick freezing in liquid N2.

### Cell culture and transfection

The human PC3 cell line was from American Type Culture Collection (ATCC) and maintained in complete RPMI 1640 supplemented with 10% fetal bovine serum (FBS). The PC3 cell lines stably expressing GFP or PTRF-GFP (PC3-GFP and PC3-GFP-PTRF) were generous gifts from Dr. Michelle Hill (The University of Queensland Diamantina Institute, Brisbane, Australia) [13]. All cell lines were passaged at least twice after recovery from frozen stocks before initiating experiments and maintained in culture for a maximum of 8 to 10 passages to minimize phenotypic drift.

Plasmid transfection was done 24 h after plating of the cells or siRNA transfection, using Lipofectamine 2000 (Life Technologies, Thermo Fisher Scientific) following the manufacturer’s protocol. Experiments were performed 24 h post plasmid transfection. In order to knockdown Cav1, cells were cultured in complete medium for 24 h before transfection with specific mouse Cav1 siRNA or control siRNA smartpools (mouse siCav1: L-0058415-00, siCONTROLS: D-001210-01; Dharmacon) using Lipofectamine 2000 transfection reagent (Life Technologies, Thermo Fisher Scientific) following the manufacturer’s protocol. For Gal3 knockdown, we used previously described custom siRNA oligonucleotide sequences and protocol [53]. Briefly, cells were transfected with the specific pool of four custom synthesized mouse Gal3 siRNA oligonucleotides duplexes or control smart pool siRNA (siCONTROLS) using Lipofectamine 2000. Cells were grown for 48 h prior to experimentation.

### Western blotting

Cell pellets from 80% confluent cultures were washed with cold PBS and resuspended in lysis buffer (20 mM Tris-HCl, pH 7.6, 0.5% NP-40, 250 mM NaCl, 3 mM EDTA, 3 mM EGTA containing freshly added 2 mM DTT, 0.5 mM PMSF, 1 mM sodium vanadate, 2.5 mM sodium fluoride, and 1 μM leupeptin) for 30 min at 4°C, pelleted at 13,000 rpm at 4°C, and the supernatant collected and stored at -80°C. Equal amounts of proteins were separated on 12% SDS-PAGE, electroblotted onto nitrocellulose (GE Healthcare Life Science), probed with indicated antibodies and HRP-conjugated secondary antibodies, and revealed by ECL (Merck Millipore).

### Immunofluorescence labeling

Cells were fixed with 3% paraformaldehyde (PFA) for 15 min at room temperature, rinsed with PBS, permeabilized with 0.1% Triton X-100 in PBS/CM, blocked with PBS/CM containing 0.2% bovine serum albumin (BSA), and then incubated with primary and fluorescent secondary antibodies in PBS/CM containing 0.2% BSA. After labeling, the coverslips were mounted in CelVol (Celanese, Ltd.), and images acquired with the 100× planapochromat objectives (NA 1.35) of an FV1000 Olympus confocal microscope.

### FRAP measurements

FRAP was performed on a confocal microscope (FV1000, Olympus) equipped with a 60x planapochromat objective (NA 1.35; oil) and SIM scanner. Cells were plated at low density on FN (10 μg/ml) for 24 h in an 8-well μ-slide chamber (ibidi), transfected with FAK-GFP or FAK-GFP plus mCherry or FAK-GFP plus PTRF-mCherry, and experiments performed 24 h later at 37°C. siRNA were transfected 48 h before FRAP experiments. Cells were treated with Gal3-His by changing the medium to serum-free medium containing indicated concentration of Gal3-His for 10 min before changing back to bicarbonate-free RPMI medium. For each FRAP analysis, a prebleach frame was acquired followed by a single bleach event using the simultaneous and independent stimulation of the 405 line of the SIM scanner. Fluorescence recovery was followed at 4-s time intervals until the intensity reached a plateau. Fluorescence during recovery was normalized to the prebleach intensity. Intensity ratios in the bleached area were compared before bleaching and after recovery to calculate mobile fractions using Prism 4 (GraphPad). Graphs are representative of a minimum of three independent experiments in which between 10 and 25 focal adhesions were bleached.

### Migration assay

For migration assay, cells were trypsinized and counted, and 200,000 cells/well were transferred to uncoated 8-μm cell culture inserts (BD Falcon) in medium containing 2% serum and the assembly placed into 24-well plates containing complete medium. After 16 h, non-migrated cells were removed from the top of the filter with a cotton swab, and migrated cells on the bottom of the filter were fixed with 3% PFA, stained with 5% crystal violet and labeled cells counted. Cell counts were normalized to the PC3 group. Alternatively, 200,000 cells/well were transferred to inserts in serum-free medium with or without 2 μg/ml Gal3-His and the assembly placed into 24-well plates containing complete medium for 14 h before fixation and staining. Cell counts were normalized to the PC3 non-treated group.

## References

[1] Parton RG, Del Pozo MA. Caveolae as plasma membrane sensors, protectors and organizers. Nature reviews Molecular cell biology. 2013;14(2):98–112. Epub 2013/01/24. 10.1038/nrm3512 23340574

[2] Navarro A, Anand-Apte B, Parat MO. A role for caveolae in cell migration. FASEB J. 2004;18(15):1801–11. Epub 2004/12/04. 15576483 10.1096/fj.04-2516rev

[3] Williams TM, Lisanti MP. Caveolin-1 in oncogenic transformation, cancer, and metastasis. American journal of physiology Cell physiology. 2005;288(3):C494–506. Epub 2005/02/05. 15692148 10.1152/ajpcell.00458.2004

[4] Goetz JG, Joshi B, Lajoie P, Strugnell SS, Scudamore T, Kojic LD, et al Concerted regulation of focal adhesion dynamics by galectin-3 and tyrosine-phosphorylated caveolin-1. J Cell Biol. 2008;180(6):1261–75. Epub 2008/03/19. 10.1083/jcb.200709019 18347068 PMC2290850

[5] Yang G, Truong LD, Timme TL, Ren C, Wheeler TM, Park SH, et al Elevated expression of caveolin is associated with prostate and breast cancer. Clinical cancer research: an official journal of the American Association for Cancer Research. 1998;4(8):1873–80. Epub 1998/08/26. 9717814

[6] Yang G, Truong LD, Wheeler TM, Thompson TC. Caveolin-1 expression in clinically confined human prostate cancer: a novel prognostic marker. Cancer Res. 1999;59(22):5719–23. Epub 1999/12/03. 10582690

[7] Gould ML, Williams G, Nicholson HD. Changes in caveolae, caveolin, and polymerase 1 and transcript release factor (PTRF) expression in prostate cancer progression. Prostate. 2010;70(15):1609–21. Epub 2010/06/22. 10.1002/pros.21195 20564315

[8] Li L, Yang G, Ebara S, Satoh T, Nasu Y, Timme TL, et al Caveolin-1 mediates testosterone-stimulated survival/clonal growth and promotes metastatic activities in prostate cancer cells. Cancer Res. 2001;61(11):4386–92. Epub 2001/06/05. 11389065

[9] Hill MM, Bastiani M, Luetterforst R, Kirkham M, Kirkham A, Nixon SJ, et al PTRF-Cavin, a conserved cytoplasmic protein required for caveola formation and function. Cell. 2008;132(1):113–24. Epub 2008/01/15. 10.1016/j.cell.2007.11.042 18191225 PMC2265257

[10] Hayer A, Stoeber M, Bissig C, Helenius A. Biogenesis of caveolae: stepwise assembly of large caveolin and cavin complexes. Traffic. 2010;11(3):361–82. Epub 2010/01/15. 10.1111/j.1600-0854.2009.01023.x 20070607

[11] Joshi B, Strugnell SS, Goetz JG, Kojic LD, Cox ME, Griffith OL, et al Phosphorylated caveolin-1 regulates Rho/ROCK-dependent focal adhesion dynamics and tumor cell migration and invasion. Cancer Res. 2008;68(20):8210–20. Epub 2008/10/17. 10.1158/0008-5472.CAN-08-0343 18922892

[12] Aung CS, Hill MM, Bastiani M, Parton RG, Parat MO. PTRF-cavin-1 expression decreases the migration of PC3 prostate cancer cells: role of matrix metalloprotease 9. Eur J Cell Biol. 2011;90(2–3):136–42. Epub 2010/08/25. 20732728 10.1016/j.ejcb.2010.06.004

[13] Inder KL, Zheng YZ, Davis MJ, Moon H, Loo D, Nguyen H, et al Expression of PTRF in PC-3 Cells modulates cholesterol dynamics and the actin cytoskeleton impacting secretion pathways. Molecular & cellular proteomics: MCP. 2012;11(2):M111 012245. Epub 2011/10/28. 10.1074/mcp.M111.012245 PMC3277761 22030351

[14] Moon H, Lee CS, Inder KL, Sharma S, Choi E, Black DM, et al PTRF/cavin-1 neutralizes non-caveolar caveolin-1 microdomains in prostate cancer. Oncogene. 2014;33(27):3561–70. Epub 2013/08/13. 10.1038/onc.2013.315 23934189

[15] Lauffenburger DA, Horwitz AF. Cell migration: a physically integrated molecular process. Cell. 1996;84(3):359–69. Epub 1996/02/09. 8608589 10.1016/s0092-8674(00)81280-5

[16] Ridley AJ, Schwartz MA, Burridge K, Firtel RA, Ginsberg MH, Borisy G, et al Cell migration: integrating signals from front to back. Science. 2003;302(5651):1704–9. Epub 2003/12/06. 14657486 10.1126/science.1092053

[17] Gupton SL, Waterman-Storer CM. Spatiotemporal feedback between actomyosin and focal-adhesion systems optimizes rapid cell migration. Cell. 2006;125(7):1361–74. Epub 2006/07/04. 16814721 10.1016/j.cell.2006.05.029

[18] Burridge K, Fath K. Focal contacts: transmembrane links between the extracellular matrix and the cytoskeleton. BioEssays: news and reviews in molecular, cellular and developmental biology. 1989;10(4):104–8. Epub 1989/04/01. 10.1002/bies.950100403 2658985

[19] Chen CS, Alonso JL, Ostuni E, Whitesides GM, Ingber DE. Cell shape provides global control of focal adhesion assembly. Biochemical and biophysical research communications. 2003;307(2):355–61. Epub 2003/07/16. 12859964 10.1016/s0006-291x(03)01165-3

[20] Giannone G, Ronde P, Gaire M, Beaudouin J, Haiech J, Ellenberg J, et al Calcium rises locally trigger focal adhesion disassembly and enhance residency of focal adhesion kinase at focal adhesions. J Biol Chem. 2004;279(27):28715–23. Epub 2004/04/23. 15102844 10.1074/jbc.M404054200

[21] Hamadi A, Bouali M, Dontenwill M, Stoeckel H, Takeda K, Ronde P. Regulation of focal adhesion dynamics and disassembly by phosphorylation of FAK at tyrosine 397. Journal of cell science. 2005;118(Pt 19):4415–25. Epub 2005/09/15. 16159962 10.1242/jcs.02565

[22] Gaus K, Le Lay S, Balasubramanian N, Schwartz MA. Integrin-mediated adhesion regulates membrane order. J Cell Biol. 2006;174(5):725–34. Epub 2006/09/01. 16943184 10.1083/jcb.200603034 PMC2064315

[23] Glenney JR Jr. Tyrosine phosphorylation of a 22-kDa protein is correlated with transformation by Rous sarcoma virus. J Biol Chem. 1989;264(34):20163–6. Epub 1989/12/05. 2479645

[24] Glenney JR Jr., Zokas L. Novel tyrosine kinase substrates from Rous sarcoma virus-transformed cells are present in the membrane skeleton. J Cell Biol. 1989;108(6):2401–8. Epub 1989/06/01. 2472406 10.1083/jcb.108.6.2401 PMC2115592

[25] Li S, Seitz R, Lisanti MP. Phosphorylation of caveolin by src tyrosine kinases. The alpha-isoform of caveolin is selectively phosphorylated by v-Src in vivo. J Biol Chem. 1996;271(7):3863–8. Epub 1996/02/16. 8632005

[26] Grande-Garcia A, Echarri A, de Rooij J, Alderson NB, Waterman-Storer CM, Valdivielso JM, et al Caveolin-1 regulates cell polarization and directional migration through Src kinase and Rho GTPases. J Cell Biol. 2007;177(4):683–94. Epub 2007/05/23. 17517963 10.1083/jcb.200701006 PMC2064213

[27] Granovsky M, Fata J, Pawling J, Muller WJ, Khokha R, Dennis JW. Suppression of tumor growth and metastasis in Mgat5-deficient mice. Nature medicine. 2000;6(3):306–12. Epub 2000/03/04. 10700233 10.1038/73163

[28] Lagana A, Goetz JG, Cheung P, Raz A, Dennis JW, Nabi IR. Galectin binding to Mgat5-modified N-glycans regulates fibronectin matrix remodeling in tumor cells. Molecular and cellular biology. 2006;26(8):3181–93. Epub 2006/04/04. 16581792 10.1128/MCB.26.8.3181-3193.2006 PMC1446937

[29] Dumic J, Dabelic S, Flogel M. Galectin-3: an open-ended story. Biochimica et biophysica acta. 2006;1760(4):616–35. Epub 2006/02/16. 16478649 10.1016/j.bbagen.2005.12.020

[30] Saravanan C, Liu FT, Gipson IK, Panjwani N. Galectin-3 promotes lamellipodia formation in epithelial cells by interacting with complex N-glycans on alpha3beta1 integrin. Journal of cell science. 2009;122(Pt 20):3684–93. Epub 2009/09/17. 10.1242/jcs.045674 19755493 PMC2758802

[31] Shankar J, Wiseman SM, Meng F, Kasaian K, Strugnell S, Mofid A, et al Coordinated expression of galectin-3 and caveolin-1 in thyroid cancer. The Journal of pathology. 2012;228(1):56–66. Epub 2012/04/20. 10.1002/path.4041 22513979

[32] Dennis JW, Nabi IR, Demetriou M. Metabolism, cell surface organization, and disease. Cell. 2009;139(7):1229–41. Epub 2010/01/13. 10.1016/j.cell.2009.12.008 20064370 PMC3065826

[33] Dennis JW, Brewer CF. Density-dependent lectin-glycan interactions as a paradigm for conditional regulation by posttranslational modifications. Molecular & cellular proteomics: MCP. 2013;12(4):913–20. Epub 2013/02/05. 23378517 10.1074/mcp.R112.026989 PMC3617338

[34] Hansen CG, Nichols BJ. Exploring the caves: cavins, caveolins and caveolae. Trends Cell Biol. 2010;20(4):177–86. Epub 2010/02/16. 10.1016/j.tcb.2010.01.005 20153650

[35] Liu L, Pilch PF. A critical role of cavin (polymerase I and transcript release factor) in caveolae formation and organization. J Biol Chem. 2008;283(7):4314–22. Epub 2007/12/07. 18056712 10.1074/jbc.M707890200

[36] Lajoie P, Goetz JG, Dennis JW, Nabi IR. Lattices, rafts, and scaffolds: domain regulation of receptor signaling at the plasma membrane. J Cell Biol. 2009;185(3):381–5. Epub 2009/04/29. 10.1083/jcb.200811059 19398762 PMC2700393

[37] Lajoie P, Kojic LD, Nim S, Li L, Dennis JW, Nabi IR. Caveolin-1 regulation of dynamin-dependent, raft-mediated endocytosis of cholera toxin-B sub-unit occurs independently of caveolae. Journal of cellular and molecular medicine. 2009;13(9B):3218–25. Epub 2009/05/15. 10.1111/j.1582-4934.2009.00732.x 19438805 PMC4516479

[38] Zheng YZ, Boscher C, Inder KL, Fairbank M, Loo D, Hill MM, et al Differential impact of caveolae and caveolin-1 scaffolds on the membrane raft proteome. Molecular & cellular proteomics: MCP. 2011;10(10):M110 007146. Epub 2011/07/15. 10.1074/mcp.M110.007146 PMC3205860 21753190

[39] Hill MM, Daud NH, Aung CS, Loo D, Martin S, Murphy S, et al Co-regulation of cell polarization and migration by caveolar proteins PTRF/Cavin-1 and caveolin-1. PLoS One. 2012;7(8):e43041 Epub 2012/08/23. 10.1371/journal.pone.0043041 22912783 PMC3418245

[40] Nassar ZD, Moon H, Duong T, Neo L, Hill MM, Francois M, et al PTRF/Cavin-1 decreases prostate cancer angiogenesis and lymphangiogenesis. Oncotarget. 2013;4(10):1844–55. Epub 2013/10/15. 24123650 10.18632/oncotarget.1300 PMC3858569

[41] Boscher C, Nabi IR. Galectin-3- and phospho-caveolin-1-dependent outside-in integrin signaling mediates the EGF motogenic response in mammary cancer cells. Mol Biol Cell. 2013;24(13):2134–45. Epub 2013/05/10. 10.1091/mbc.E13-02-0095 23657817 PMC3694797

[42] Nomura R, Fujimoto T. Tyrosine-phosphorylated caveolin-1: immunolocalization and molecular characterization. Mol Biol Cell. 1999;10(4):975–86. Epub 1999/04/10. 10198051 10.1091/mbc.10.4.975 PMC25222

[43] Parton RG, Hanzal-Bayer M, Hancock JF. Biogenesis of caveolae: a structural model for caveolin-induced domain formation. Journal of cell science. 2006;119(Pt 5):787–96. Epub 2006/02/24. 16495479 10.1242/jcs.02853

[44] Demetriou M, Nabi IR, Coppolino M, Dedhar S, Dennis JW. Reduced contact-inhibition and substratum adhesion in epithelial cells expressing GlcNAc-transferase V. J Cell Biol. 1995;130(2):383–92. Epub 1995/07/01. 7615638 10.1083/jcb.130.2.383 PMC2199932

[45] Wei Y, Yang X, Liu Q, Wilkins JA, Chapman HA. A role for caveolin and the urokinase receptor in integrin-mediated adhesion and signaling. J Cell Biol. 1999;144(6):1285–94. Epub 1999/03/24. 10087270 10.1083/jcb.144.6.1285 PMC2150580

[46] del Pozo MA, Balasubramanian N, Alderson NB, Kiosses WB, Grande-Garcia A, Anderson RG, et al Phospho-caveolin-1 mediates integrin-regulated membrane domain internalization. Nat Cell Biol. 2005;7(9):901–8. Epub 2005/08/23. 16113676 10.1038/ncb1293 PMC1351395

[47] Radel C, Rizzo V. Integrin mechanotransduction stimulates caveolin-1 phosphorylation and recruitment of Csk to mediate actin reorganization. Am J Physiol Heart Circ Physiol. 2005;288(2):H936–45. Epub 2004/10/09. 15471980 10.1152/ajpheart.00519.2004

[48] Fukumori T, Oka N, Takenaka Y, Nangia-Makker P, Elsamman E, Kasai T, et al Galectin-3 regulates mitochondrial stability and antiapoptotic function in response to anticancer drug in prostate cancer. Cancer Res. 2006;66(6):3114–9. Epub 2006/03/17. 16540661 10.1158/0008-5472.CAN-05-3750

[49] Wang Y, Nangia-Makker P, Balan V, Hogan V, Raz A. Calpain activation through galectin-3 inhibition sensitizes prostate cancer cells to cisplatin treatment. Cell death & disease. 2010;1:e101. Epub 2010/01/01. 21368866 10.1038/cddis.2010.79 PMC3032324

[50] Wang Y, Balan V, Gao X, Reddy PG, Kho D, Tait L, et al The significance of galectin-3 as a new basal cell marker in prostate cancer. Cell death & disease. 2013;4:e753. Epub 2013/08/03. 23907467 10.1038/cddis.2013.277 PMC3763439

[51] Glinskii OV, Sud S, Mossine VV, Mawhinney TP, Anthony DC, Glinsky GV, et al Inhibition of prostate cancer bone metastasis by synthetic TF antigen mimic/galectin-3 inhibitor lactulose-L-leucine. Neoplasia. 2012;14(1):65–73. Epub 2012/02/23. 22355275 10.1593/neo.111544 PMC3281943

[52] Guha P, Kaptan E, Bandyopadhyaya G, Kaczanowska S, Davila E, Thompson K, et al Cod glycopeptide with picomolar affinity to galectin-3 suppresses T-cell apoptosis and prostate cancer metastasis. Proceedings of the National Academy of Sciences of the United States of America. 2013;110(13):5052–7. Epub 2013/03/13. 10.1073/pnas.1202653110 23479624 PMC3612646

[53] Henderson NC, Mackinnon AC, Farnworth SL, Poirier F, Russo FP, Iredale JP, et al Galectin-3 regulates myofibroblast activation and hepatic fibrosis. Proceedings of the National Academy of Sciences of the United States of America. 2006;103(13):5060–5. Epub 2006/03/22. 16549783 10.1073/pnas.0511167103 PMC1458794

[54] Wallner M, Gruber P, Radauer C, Maderegger B, Susani M, Hoffmann-Sommergruber K, et al Lab scale and medium scale production of recombinant allergens in Escherichia coli. Methods. 2004;32(3):219–26. Epub 2004/02/14. 14962755 10.1016/j.ymeth.2003.08.004

